# Whole-genome Sequencing for Tracing the Transmission Link between Two ARD Outbreaks Caused by a Novel HAdV Serotype 7 Variant, China

**DOI:** 10.1038/srep13617

**Published:** 2015-09-04

**Authors:** Shaofu Qiu, Peng Li, Hongbo Liu, Yong Wang, Nan Liu, Chengyi Li, Shenlong Li, Ming Li, Zhengjie Jiang, Huandong Sun, Ying Li, Jing Xie, Chaojie Yang, Jian Wang, Hao Li, Shengjie Yi, Zhihao Wu, Leili Jia, Ligui Wang, Rongzhang Hao, Yansong Sun, Liuyu Huang, Hui Ma, Zhengquan Yuan, Hongbin Song

**Affiliations:** 1 Institute of Disease Control and Prevention, Academy of Military Medical Sciences, Beijing 100071, China; 2 The No. 477 Hospital of PLA, Xiangyang 441003, China; 3 Air Force Center for Disease Control and Prevention, Beijing 100076, China; 4 Health Department of General Logistics Department, PLA, 22 Fuxing Road, Beijing 100842, China

## Abstract

From December 2012 to February 2013, two outbreaks of acute respiratory disease caused by HAdV-7 were reported in China. We investigated possible transmission links between these two seemingly unrelated outbreaks by integration of epidemiological and whole-genome sequencing (WGS) data. WGS analyses showed that the HAdV-7 isolates from the two outbreaks were genetically indistinguishable; however, a 12 bp deletion in the virus-associated RNA gene distinguished the outbreak isolates from other HAdV-7 isolates. Outbreak HAdV-7 isolates demonstrated increased viral replication compared to non-outbreak associated HAdV-7 isolate. Epidemiological data supported that the first outbreak was caused by introduction of the novel HAdV-7 virus by an infected recruit upon arrival at the training base. Nosocomial transmission by close contacts was the most likely source leading to onset of the second HAdV-7 outbreak, establishing the apparent transmission link between the outbreaks. Our findings imply that in-hospital contact investigations should be encouraged to reduce or interrupt further spread of infectious agents when treating outbreak cases, and WGS can provide useful information guiding infection-control interventions.

Human adenoviruses (HAdVs) are non-enveloped, icosahedral, double-stranded DNA viruses that are classified into seven species (A–G) based on their nucleotide homology, haemagglutination, biochemical and biological properties; at least 51 serotypes are recognized^1^,^2^,^3^. HAdV can cause a wide range of clinical syndromes, including respiratory tract illnesses, conjunctivitis, cystitis, gastroenteritis, neurological diseases; in some cases infection directly results in death^1^,^4^. HAdV is reported to be one of the primary causes of acute respiratory disease (ARD) worldwide, which is responsible for 5–10% of lower respiratory tract infections in children throughout the world^2^,^5^. Serotype 7 (HAdV-7) accounts for nearly 20% of all HAdV infections reported to the World Health Organization^4^,^6^, and is more frequently associated with severe illnesses, particularly in children aged <7 years and persons with underlying health conditions^7^,^8^. Moreover, HAdV-7 is among the most prevalent serotypes associated with ARD outbreaks in institutional, hospital and community settings^2^,^4^,^7^.

Whole-genome sequencing (WGS) has recently been applied to rapidly discriminate outbreaks caused by bacteria such as *Escherichia coli* O104: H4^9^, methicillin-resistant *Staphylococcus aureus*^10^,^11^, *Mycobacterium tuberculosis*^12^,^13^,^14^, carbapenem-resistant *Klebsiella pneumoniae*^15^ and *Legionella pneumophila*^16^, but has been used with limited study size or scope in describing transmission links during the course of outbreaks caused by viruses. From December 2012 to February 2013, two ARD outbreaks caused by HAdV-7 occurred at two training bases located in different cities (separated by approximately 170 km) in Hubei Province, China. A total of 946 persons were symptomatic as part of these two outbreaks. An investigation of these two HAdV-7 outbreaks to determine whether a potential transmission link could be identified is described using WGS coupled with clinical and epidemiological investigations.

## Results

### Overview of the outbreaks

**Figure 1:**
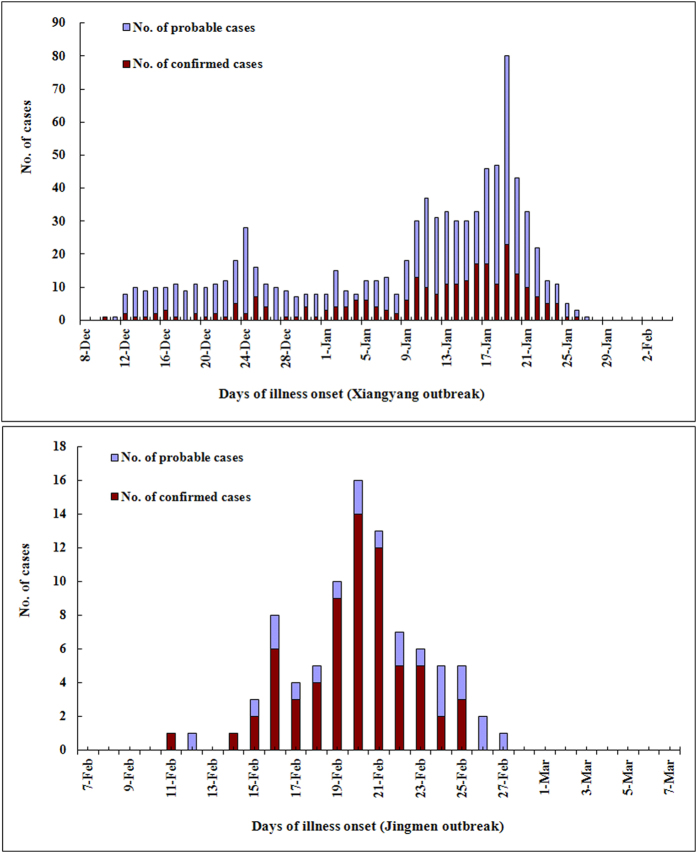
Cases of confirmed and probable HAdV-7 infections during the two outbreaks. A total of 946 persons were affected during the outbreaks. We collected 328 and 80 nasopharyngeal specimens from the two outbreak patients, respectively, and 323 HAdV-7 isolates were identified by PCR assays and viral culture.

Base A (Xiangyang) houses 1290 recruits, of whom 858 (66.5%) fell ill with ARD from December 10, 2012 to January 28, 2013 (Fig. 1). Among the 858 cases, 321 (37.4%) were hospitalized, 108 (12.6%) developed acute pneumonia and 37 were considered severe cases. Base B (Jingmen) houses 865 recruits; 88 (10.2%) were diagnosed with ARD from February 11 to 27, 2013. Among the 88 cases, 42 (47.7%) were hospitalized, 16 (18.2%) with pneumonia and 4 severe cases were recorded. Under normal circumstances, these training facilities are not directly connected; this is primarily due to the geographic separation (Figure S1) and self-contained management systems. The initial hypothesis was that these outbreaks were unrelated.

The mean ages of the cases at the onset of illness were 19.7 and 20.1 years, respectively (Table S5). A small number of cases from both outbreaks had underlying medical conditions; the most common condition was allergy to antibiotics (6.1% & 8.0%). Analysis of clinical characteristics of the hospitalized cases from the two outbreaks showed very similar clinical features (Table 1). A total of 36.2% and 38.1% of the hospitalized cases from the outbreaks showed acute pneumonia. Among the pneumonia cases, more than 50% had bilateral pneumonia, and 34.3% and 25.0% of them developed into severe cases, respectively. Hospitalized cases from the outbreaks also showed very similar clinical laboratory and radiologic findings, and abnormal findings such as abnormalities on chest radiograph and CT were commonly observed among the hospitalized cases from the outbreaks (Table 2). These abnormal manifestations may be indicators of multiorgan involvement among the cases^17^.

### Epidemiologic investigations of the outbreaks

**Figure 2:**
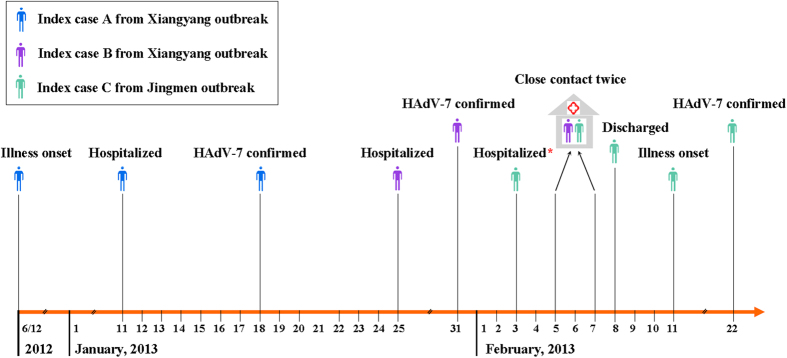
Timeline of critical events and nosocomial transmission for the index cases. Index case C was hospitalized due to a training injury from February 3 to 8, 2013. During his hospitalization, there were also about 70 patients hospitalized in the Xiangyang affiliated hospital from the Base A outbreak (Xiangyang). All cases were housed in the same in-patient building. Index case C had close contact with Index case B, a confirmed HAdV-7 case from the Xiangyang outbreak. They reported verbal contact twice for about half an hour on February 5 and 7, 2013, respectively. After 5 days of hospitalization, he returned to the training base B (Jingmen) on February 8, 2013, and took part in the normal training without isolation for medical observation. On February 11, 2013, he developed symptoms of ARD with fever (maximum temperature 39·0 °C), sore throat, cough and headache. A nasopharyngeal sample collected from this patient was detected positive for HAdV-7 by PCR sequencing on February 22, 2013. Thereafter, 5 roommates consecutively had ARD symptoms. These investigations revealed that nosocomial transmission by close contact contributed to the introduction of HAdV-7 into the training base B (Jingmen), indicating the potential transmission link between the two outbreaks. This figure including the drawings of people was drawn by the author Shaofu Qiu.

Based on the similar demographic characteristics, clinical presentations, laboratory and radiographic findings, possible epidemiological associations that might link the outbreaks was explored. Preliminary investigations identified a 22-year-old male recruit (Index case A) who had ARD symptoms before reporting to Base A (Xiangyang) on December 6, 2012 (Fig. 2). He arrived at the training base on December 10, 2012. During the next one month, he was treated in the clinic of Base A by empirical administration of roxithromycin, lincomycin, ceftriaxone and ribavirin, however his condition did not resolve and by January 10, 2013 had worsened to persistent high fever (39–39.9 °C) and shortness of breath. The case was admitted to Xiangyang affiliated hospital on January 11, 2013 where he was diagnosed as a severe pneumonia case with multiorgan damage. He had an elevated WBC count (12.5 × 10^9^ cells/liter), creatine kinase (56867 U/liter), lactate dehydrogenase (348 U/liter), aspartate aminotransferase (687 U/liter), alanine aminotransferase (303 U/liter) and C-reactive protein (28.16 mg/liter), and decreased level of potassium (3.39 μmol/liter). He was treated with additional antimicrobial and antiviral agents including azithromycin, levofloxacin, cefepime and ribavirin, and methylprednisolone was intravenously administered (range, 40–320 mg per day). Liver-protective and myocardial nutritional drugs were also prescribed. After 25 days of hospitalization the case made a full recovery. A serum sample collected on January 10, 2013 was positive for HAdV IgA, and a HAdV-7 isolate was identified from a nasopharyngeal sample collected on January 17, 2013 by PCR and viral culture, suggesting that the case had prolonged shedding of adenovirus (Fig. 2). From December to January, this case was not quarantined at Base A, and he participated in normal training and educational activities including the training course opening ceremony on December 20, 2012. During this period, 9 recruits living in the same dormitory (9/12) with the index case successively showed ARD symptoms; HAdV-7 was detected in 7 (77.8%) by PCR analysis of nasopharyngeal samples.

During our investigation, an observation that cases unable to be diagnosed and/or treated in the medical clinics associated with Base A and B were usually transferred to Xiangyang affiliated hospital was made. Importantly, while the outbreak was occurring at Base A, during which time >300 cases were sent to the Xiangyang affiliated hospital in one month, a 19-year-old male recruit from base B (Jingmen) (Index case C) had been sent for treatment to Xiangyang affiliated hospital for lumbago and backpain on February 3, 2013 (Fig. 2). Physicians did not strictly comply with infection control measures established by the hospital; primarily because of poor awareness of infection control for the inpatients. While Index case C was receiving treatment, he reported having two, half an hour conversations with an acquaintance from Base A (Index case B) who was hospitalized for ARD (Fig. 2). After returning to Base B, Index Case C participated in routine training exercises but developed ARD on February 11, 2013. Infection with HAdV-7 was confirmed by PCR and subsequent DNA sequence analysis; five roommates of Index case C subsequently showed ARD symptoms.

### Laboratory investigations and genome sequencing

A total of 377 HAdV-7 isolates were identified by PCR and viral culture; 323 were from outbreak cases including the three index cases, 48 were from close contacts to these cases, and 6 were from non-recruit local ARD patients. The positive rates of HAdV-7 for the outbreak cases, close contacts and non-recruit ARD cases were 79.2%, 30.0% and 2.6%, respectively (Figure S2). Among the non-recruit cases, HAdV-7 was identified from four cases from Xiangyang City and two from Jingmen City. Phylogenetic analysis based on whole genes extracted from sequenced genomes showed that the HAdV-7 isolates from the outbreak cases, close contacts and non-recruit cases were indistinguishable in the hexon and fiber genes.

**Figure 3:**
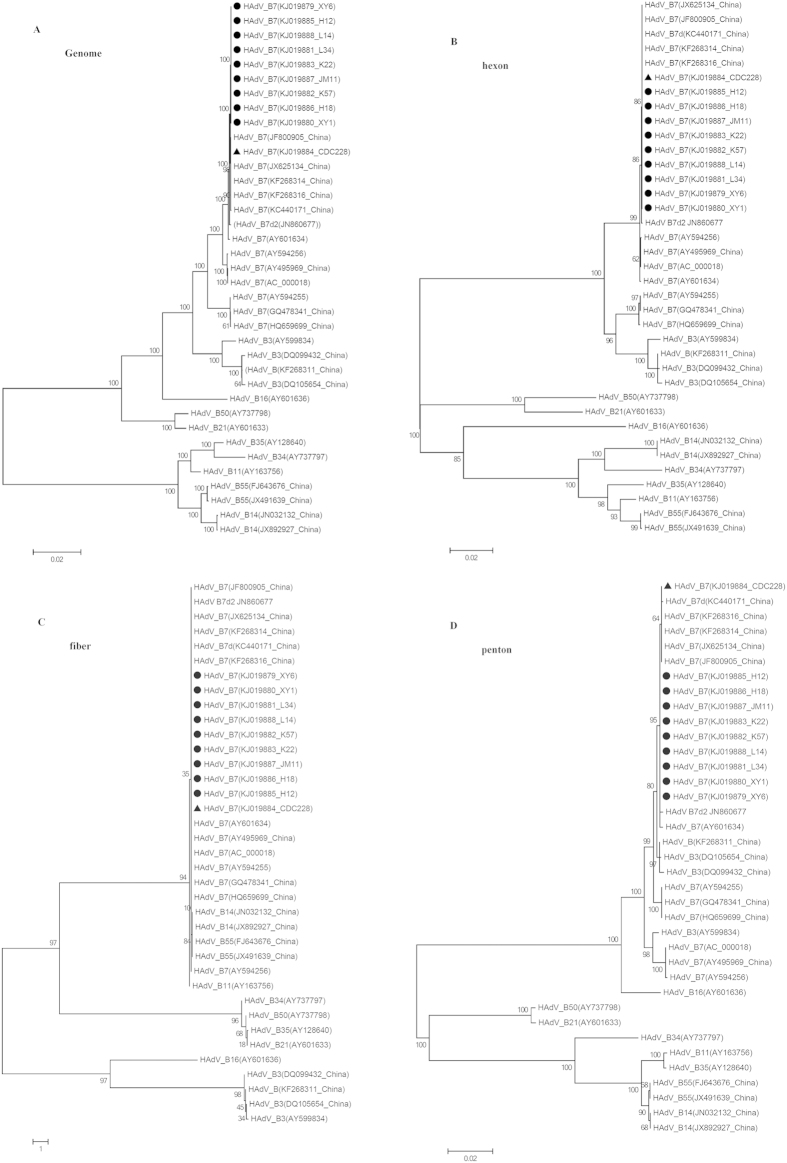
Phylogenetic analysis of the outbreak isolates based on WGS and individual genes. Panels (**A**–**D**) represent the phylogenetic trees of the whole-genome, and individual entire hexon, fiber and penton genes, respectively. Outbreak strains are highlighted in circle (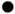), and the local strain CDC 228 from Xiangyang City is indicated in triangle (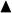). Bootstrap analysis was performed with 1,000 replicates. Representative HAdV strains with GenBank accession numbers are included in the phylogenetic trees, and they are also shown in Table S2.

**Figure 4:**
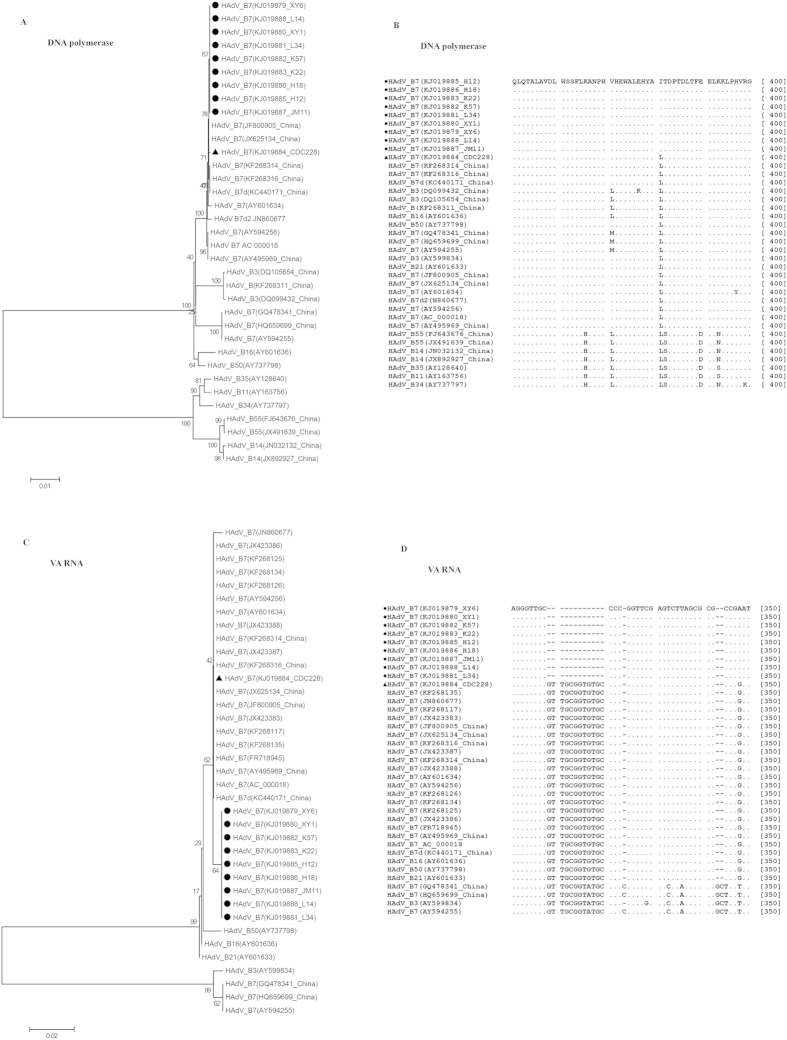
Phylogenetic analysis and pairwise alignment of the outbreak isolates based on DNA polymerase and VA RNA. Panels (**A**,**C**) represent the phylogenetic trees of DNA polymerase and VA RNA genes, respectively. Panels (**B**,**D**) represent the pairwise alignment analyses of the outbreak strains based on the DNA polymerase amino acid sequences and VA RNA genes, respectively.

To rapidly measure the degree of genetic relatedness among the HAdV-7 isolates from the outbreak and non-recruit cases, WGS was performed on ten HAdV-7 isolates including those from the index cases. Genome sequences were deposited in the GenBank database (accession nos. KJ019879-KJ019888). By aligning the sequenced genomes and using 0901 HZ as a reference genome (accession no. JF800905), the nine outbreak isolates, including those from the index cases (accession nos. KJ019879, KJ019880 and KJ019885), were 100% identical. Phylogenetic analysis of the WGS showed that the outbreak isolates formed an independent sublineage among the HAdV-7 strains (Fig. 3A). Comparative genomic and phylogenetic analyses showed that the outbreak isolates had genomes nearly identical with an isolate CDC228 (accession no. KJ019884) identified from a local patient, as well as other five strains (accession nos. JF800905, KC440171, JX625134, KF268314 and KF268316) circulating in China^6^,^18^,^19^. Further phylogenetic analysis of the individual whole genes revealed that the outbreak isolates clustered with the local isolate CDC228 as well as the above five strains, and shared 100% similarities in the hexon and fiber genes (Fig. 3B,C). However, the outbreak strains formed a separate subclade in the phylogenetic trees based on the penton, DNA polymerase and VA RNA genes (Figs 3D and 4A,C). Genome comparisons revealed that the outbreak isolates had only minor differences in the penton and DNA polymerase genes with CDC228 and the above reference strains (Table 3, Figs 3D and 4A), but a distinct point mutation was observed among them when compared with other HAdV-7 strains, resulting in the replacement of Leu381 with Ile (Table 3 and Fig. 4B). Additionally, all of the outbreak isolates showed an obvious difference with other HAdV-7 strains in the VA RNA genes (Fig. 4C), with a unique deletion of 12 base pair in the VA RNA II region (Table 3 and Fig. 4D). The universality of this unique deletion and the distinct point mutation were verified among an additional 46 HAdV-7 outbreak strains by PCR and DNA sequencing (data not shown).

### Growth kinetics of viral replication

**Figure 5:**
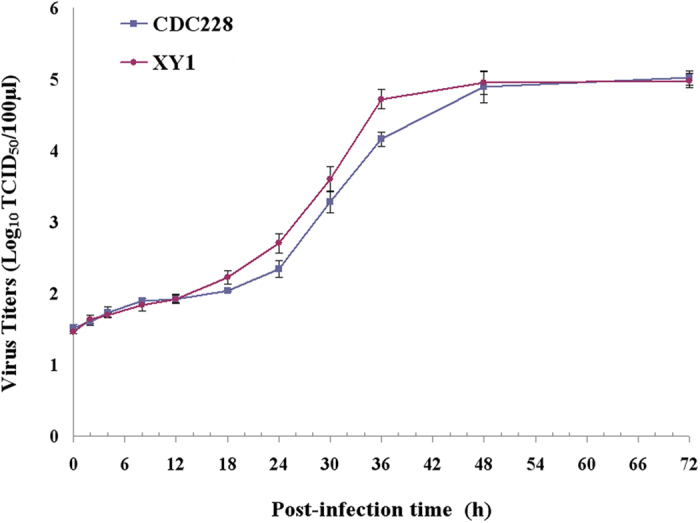
Replication kinetic of a representative outbreak-associated isolate XY1 relative to non-outbreak-associated isolate CDC228. Since the outbreak isolates shared an indistinguishable genome content, a representative outbreak-associated isolate XY1 was used to evaluate the viral replication. The isolate XY1 was recovered from the first case (Index case A) from the Xiangyang outbreak, and the on-outbreak-associated isolate CDC228 was recovered from a sporadic ARD patient from Xiangyang City. Virus titers were determined in Hep-2 cells by measuring the 50% tissue culture infectious dose (TCID_50_).

The *in vitro* growth kinetics of viral replication were examined using isolate XY1 as representative of the outbreak-associated HAdV-7 viruses. Similar growth kinetics were observed with the non-outbreak associated isolate CDC228 between 0–12 h post-infection, while XY1 had a reproducibly higher viral titer than that of CDC228 between 12–48 h post-infection, indicating enhanced viral replication of outbreak isolates during this time period (Fig. 5).

## Discussion

In China, HAdVs are not notifiable infectious pathogens, and large-scale epidemiological data from HAdV infections nationwide is currently not available. According to literature published from 1983 to 2013, at least 30 HAdV outbreaks have been reported in China, most of these have occurred since the year 2000^6^,^20^,^21^,^22^,^23^,^24^,^25^,^26^,^27^,^28^,^29^,^30^,^31^. To date, at least ten HAdV-7 outbreaks in China have been described, 5 were reported before 1993^32^,^33^,^34^,^35^, one in 2009^6^, one in 2011^19^, one in 2012^28^ and two described in this study. Interestingly, the HAdV-7 outbreaks occurring before 1993 were mainly associated with pharyngoconjunctival fever symptoms in children; no deaths were reported^32^,^33^,^34^,^35^. However, recently reported HAdV-7 outbreaks were all associated with symptoms of ARD, sometimes leading to severe or fatal illnesses in otherwise healthy persons living in China^6^,^28^. In this study the two HAdV-7 outbreaks mainly infected the healthy young adults, and serious or even life-threatening infections were often observed; fortunately no deaths were recorded. Several distinct clinical characteristics were observed from cases comprising the two outbreaks reported in this study. Most of the hospitalized patients with sputum production had yellow sputum (62.0% & 70.6%), and leukocytosis (WBC count, >10.0 × 10^9^ cells/liter) was more frequently seen in hospitalized patients (Table 2). These characteristics were notable differences from those described in the outbreaks associated with other HAdVs or other respiratory viruses^36^,^37^,^38^. Moreover, the high attack rate (66.5%) observed during the Base A (Xiangyang) outbreak places this outbreak as one of the largest HAdV-7 outbreaks documented worldwide^2^. These observations imply that virulence associated with HAdV is changing in China; this may be due to the emergence of more virulent new genotypes within existing serotypes, host factors, or a combination of these possibilities.

Combining the clinical observations, laboratory studies, epidemiological investigations and WGS analyses, the first case (Index case A) was identified as a recruit whose home was Hunan not Hubei Province. Distinct genetic differences were documented between the genomes of outbreak-associated and circulating, non-outbreak associated HAdV-7 isolates. WGS analysis strongly supported the hypothesis that the outbreak was caused by an imported HAdV-7 isolate. We were not able to investigate the prevalence of HAdV-7 in other regions of China, especially that in Hunan Province, therefore we were unable to determine the exact source of the HAdV-7 outbreak isolate. A significant contributor to the dispersal of the virus and extension of the outbreak was the lack of awareness of infectious disease control and the absence of properly trained medical personnel at training base A. Index case A, who had ARD symptoms in early December 2012, was not quarantined during the time of his treatment at Base A. This patient was possibly a super-spreader as he probably had prolonged shedding of adenovirus. Prolonged shedding of adenovirus and its hardy nature are therefore conducive to the dissemination of adenovirus to others especially the people in close contact^6^. To effectively prevent and control the occurrence of infectious disease outbreaks in the schools and training institutions especially when freshmen or new recruits enrolled, strengthening of pathogen surveillance and quarantine inspection to rapidly find and timely investigate potential transmission is critical.

This study has clearly demonstrated supplementing traditional outbreak investigations with WGS analysis provides a powerful discriminatory method for identifying transmission links between apparently unrelated outbreaks. In this case, WGS clearly established the role of nosocomial infection in the transmission and introduction of HAdV-7 into a geographically unlinked location. Without WGS analysis, it is likely that nosocomial transmission of HAdV-7 among patients in the hospital would not have been appreciated, leading to the false hypothesis that the two outbreaks were independent. Further, the relationship between the outbreak and circulating non-outbreak-associated isolates in the greater community became clear using WGS analysis. In the absence of WGS, endemic circulating HAdV isolates most likely would have been mistakenly identified as outbreak isolates because both isolates were indistinguishable using traditional genotyping based on the sequences of hexon and fiber genes. WGS revealed that HAdV-7 isolates from the two outbreaks were genetically indistinguishable, while could be distinguished by small but significant changes, with a non-synonymous substitution in DNA polymerase gene and a unique 12 bp deletion in the VA RNA II region when compared to endemic HAdV-7 isolates in China. This finding, along with the epidemiological picture, identified the most likely transmission link between the two outbreaks.

Epidemiological investigation revealed that Xiangyang affiliated hospital played a critical role in the continuation and further transmission of the outbreak. This study identified areas of patient management and infection control that were subsequently strengthened at the hospital as well as the medical clinics at Base A and B, including contact tracing investigations and infection-control interventions to reduce the risk of further transmission, infection and associated morbidity. As a result, rapid and effective control of both outbreaks occurred. Ironically, hospitals remain a central problem of inadvertent infections, since they provide a suitable ecosystem in which a variety of pathogens can emerge and persist^39^, possibly resulting in serious life-threatening infections and significant hospital-associated outbreaks^15^. Pathogen transmission in hospitals and the transmission from hospitals to the community must be recognized as a major driving force for infectious disease epidemics or outbreaks, and more attention should be paid to prevent their potential as a public health threat.

Another major finding of this study is the unexpected identification of a novel HAdV-7 variant by WGS. Genetic variation can greatly drive the emergence of new pathogens or novel variants, resulting in multidrug resistance, and increased adaptation, transmissibility and virulence. New pathogens or novel variants have in more recent years been more frequently detected, seriously threatening public health on both local and global scales. Recent examples illustrating this point are provided by the 2009 influenza pandemic caused by the 2009 H1N1 virus^40^, the worldwide concern about antimicrobial resistance posed by NDM-1 producing bacteria^41^, and the large outbreak of haemolytic uraemic syndrome caused by a strain of *E. coli* O104:H4^42^. In particular, China is one of the largest reservoirs for the emergence of new pathogens or novel variants, such as the SARS coronavirus in 2003, a novel bunyavirus in 2011^43^, and a novel avian-origin influenza A (H7N9) virus in 2013^44^. In recent years, novel species or variants of HAdVs have been commonly reported, with enhanced virulence and ability of cross-species transmission^45^,^46^,^47^. Growth kinetics revealed that the outbreak-associated HAdV-7 isolate seemingly had higher viral replication than the local HAdV-7 isolate. It would be interesting to determine what impact the 12 bp deletion in VA RNA II gene has on viral pathogenicity. Continuous surveillance is urgently required to explore the spatiotemporal variation and evolution of HAdVs.

In conclusion, WGS has potential for use in aiding outbreak investigations given its enhanced discriminatory capacity, including identifying the known and emerging pathogens, analyzing the variation and evolution, determining the origins and transmission dynamics of the outbreaks, and informing appropriate infection-control interventions^39^,^48^. In this study the definite transmission link between two ARD outbreaks caused by a novel HAdV-7 variant by integration of genomic and epidemiological data was demonstrated. HAdV outbreaks have been increasingly reported in recent years in China, concomitant with the variation and evolution of HAdVs, more attention should be given to the potential threat on public health, and strengthen the nationwide and systematic surveillance of HAdVs.

## Methods

### Outbreak investigations

From December 10, 2012 to January 28, 2013, an outbreak of ARD occurred in a recruit training base (Base A) located in Xiangyang City, Hubei Province. A second ARD outbreak was reported in a different training base (Base B), located in Jingmen City, Hubei Province from February 11 to 27, 2013 (Figure S1). Two field investigation teams, comprised of health officials, epidemiologists, technicians and medical staff, were dispatched to investigate each outbreak. Demographic and epidemiologic data were collected through interviews and field observations using a standardized survey. Medical records of hospitalized patients were abstracted by physicians who had been taking care of cases; these were copied and provided to the investigation teams. A probable or confirmed case with HAdV-7 infection were defined as previously described^49^,^50^. A close contact was defined as a person that has closely contacted with the suspected or confirmed cases within 8 days; close contacts without ARD symptoms were quarantined for medical observation for 8 days. We defined a severe case as a patient who was admitted to an ICU for serious complications, such as pneumonia, heart and liver failure, shock, and sepsis^27^,^51^. Mild cases were reviewed by trained physicians, quarantined and treated in the training base; moderate or severe cases were transferred to Xiangyang affiliated hospital in the Xiangyang City, Hubei Province.

### Laboratory investigation

A total of 796 nasopharyngeal specimens were collected during the outbreak period; 328 from Base A (Xiangyang City) and 80 from Base B (Jingmen City) outbreak patients, 160 from the close contacts including 89 from Base A and 71 from Base B, and 228 from non-recruit patients diagnosed with ARD in other hospitals in the local consisting of 128 from Xiangyang and 100 from Jingmen. Blood and sputum samples from severe cases were also collected and analyzed for possible bacterial infections using biochemcial test kits (API, bioMerieux, Marcy l’etoile, France) and 16S rRNA sequencing (Table S1). Additionally, 135 sera samples were obtained from the new recruits of training base A, which were collected for medical examination of the new recruits by the Xiangyang affiliated hospital when they reported on December 10, 2012. Specimens were sent to the Institute of Disease Control and Prevention, Academy of Military Medical Sciences (Beijing) central laboratory for identification and confirmation. For the nasopharyngeal samples collected in the early outbreak, multiplex PCR assays, Seeplex® RV12 ACE and Seeplex® Pneumabacter detection kits (Seegene, Seoul, Korea) were used to screen the suspected pathogens including Adenovirus, Coronavirus, Parainfluenza virus, Influenza A virus, Influenza B virus, Respiratory syncytial virus A, *Bordetella pertussis*, *Legionella pneumophila*, *Chlamydophila pneumoniae*, *Streptococcus pneumoniae*, *Haemophilus influenzae*, and *Mycoplasma pneumonia*. A commercial HAdV real-time PCR kit (BioPerfectus Technologies, Jiangsu, China) was used to detect virus from all of the nasopharyngeal samples. ELISA classic adenovirus kits (Institute Virion/Serion GmbH, Würzburg, Germany) were used to detect IgA and IgG antibodies against all serotypes of HAdV from sera samples. Viral culture was performed using Hep-2 cells^6^,^27^. Specimens positive for HAdVs were subtyped after direct sequencing the PCR amplified products of partial segments of viral hexon, fiber and virus-associated RNA (VA RNA) genes using primers either described previously or designed as a part of this study (Table S1). Viral growth kinetics were based on a study performed with outbreak-associated representative strain XY1, isolated from the Index case A from Base A, which was measured and compared with the HAdV-7 strain CDC228, recovered from a non-outbreak associated case, as described previously^52^,^53^.

### *De novo* genome sequencing and phylogenetic analysis

Ten representative HAdV-7 isolates, including one from a non-recruit ARD case in Xiangyang City, 4 from Base A (Xiangyang) outbreak cases and 5 from Base B (Jingmen) outbreak cases (Table S2), were selected and submitted for *de novo* WGS using a MiSeq system (Illumina, San Diego, CA, USA). Virus DNAs were extracted from the nasopharyngeal specimens using the QIAamp MinElute Virus Spin Kit (Qiagen) according to the manufacture’s instruction and randomly fragmented to an average size of 350 bp by using a Covaris S200 system (Covaris Inc). Sequencing libraries were then prepared by using the TruSeq standard DNA preparation protocol (Illumina, San Diego, CA, USA). Paired-end 2 × 150 bp sequencing was performed on a Illumina MiSeq system. After filtering low quality bases and trimming of the adapter, *De novo* assemblies were performed using Velvet 1.2.08 and VelvetOptimiser 2.24 with k values of between 21 and 151 for searching maximal N50 and the largest contig value. Sanger sequencing was used to close gaps and verify ambiguous sequences with primers listed in Table S3, designed by aligning with a reference genome 0901HZ (JF800905). Information on the number of reads sequenced, the number of viral reads and the depth of coverage of the virus was provided in Table S4. Genome annotations were completed based on the annotation of 0901HZ. Comparative genomic analysis was performed to find nucleotide acid changes and amino acid substitutions between the outbreak strains and the local or other HAdV-7 strains circulating in China. The 12-bp deletion in the VA RNA gene and the point mutation in DNA polymerase gene were verified by screening an additional 46 HAdV-7 outbreak strains by PCR and DNA sequencing with primers listed in Table S1. Multiple-sequence alignments were conducted using Clustalw2. Thirty-seven additional sequences of HAdVs were retrieved from GenBank (Table S2), and neighbor-joining phylogenic trees with 1000 bootstrap replicates were constructed based on WGS and individual gene sequences by using the MEGA software version 6.06.

All experimental protocols were approved by the Medical Ethics Committee of the Academy of Military Medical Sciences. The methods were carried out in accordance with the approved guidelines. Written informed consents for the use of their clinical samples were obtained from the corresponding patients.

## Additional Information

**How to cite this article**: Qiu, S. *et al.* Whole-genome Sequencing for Tracing the Transmission Link between Two ARD Outbreaks Caused by a Novel HAdV Serotype 7 Variant, China. *Sci. Rep.* **5**, 13617; doi: 10.1038/srep13617 (2015).

## Supplementary Material

Supplementary Information

## Figures and Tables

**Table 1:** Clinical characteristics of the hospitalized patients from the two outbreaks#.

**Characteristic**	Value*	
	**Xiangyang outbreak (n = 321)**	**Jingmen outbreak (n = 42)**
Symptoms or signs		
Fever		
Any-no. (%)	319 (99.4)	42 (100.0)
Subgroup-no. (%)		
37.3–38.0 °C	10 (3.1)	0
38.1–39.0 °C	131 (40.8)	20 (47.6)
>39.0 °C	178 (55.5)	22 (52.4)
Maximal temperature (°C), mean	39.2 ± 0.6	39.4 ± 0.7
Duration of fever, median (range), days	4.0 (1–20)	4.0 (2–11)
Cough	304 (94.7)	40 (95.2)
Throat congestion	296 (92.2)	36 (85.7)
Sore throat	246 (76.6)	32 (76.2)
Sputum production -no./total no. (%)	234/321 (72.9)	34/42 (81.0)
Yellow sputum	145/234 (62.0)	24/34 (70.6)
White sputum	89/234 (38.0)	10/34 (29.4)
Blood-streaked sputum	28/234 (12.0)	6/34 (17.6)
Swelling of tonsils	211 (65.7)	24 (57.1)
Fatigue	139 (43.3)	21 (50.0)
Pharyngeal lymphoid follicular hyperplasia	126 (39.3)	15 (35.7)
Purulent exudate on the tonsil	102 (31.8)	12 (28.6)
Rhinorrhea	79 (24.6)	9 (21.4)
Myalgia	76 (23.7)	8 (19.0)
Headache	64 (19.9)	8 (19.0)
Dizziness	61 (19.0)	5 (11.9)
Shortness of breath	54 (16.8)	8 (19.0)
Chest pain	51 (15.9)	9 (21.4)
Diarrhea	30 (9.3)	5 (11.9)
Nasal congestion	29 (9.0)	2 (4.8)
Chill	23 (7.2)	2 (4.8)
Enlargement of lymph nodes	20 (6.2)	4 (9.5)
Abdominal pain	19 (5.9)	3 (7.1)
Bronchitis	18 (5.6)	1 (2.4)
Nausea	12 (3.7)	5 (11.9)
Vomiting	8 (2.5)	1 (2.4)
Palpitation	3 (0.9)	1 (2.4)
Submandibular lymphadenitis	3 (0.9)	0
Tinnitus, earache	3 (0.9)	0
Skin rash	2 (0.6)	1 (2.4)
Convulsion, coma	2 (0.6)	0
Testicular pain	2 (0.6)	0
Clinical outcome		
Hospitalization, no./total no. (%)	321/858 (37.4)	42/88 (47.7)
Time from illness onset to hospitalization, median (range), days	3.0 (1–36)	3.0 (1–7)
Duration of hospitalization, median (range), days	10.0 (6–25)	9.0 (7–25)
Pneumonia, no./total no. (%)	108/321 (36.2)	16/42 (38.1)
Admission to an intensive care unit	37/108 (34.3)	4/16 (25.0)
Heart injury	14/108 (13.0)	2/16 (12.5)
Liver injury	11/108 (10.2)	3/16 (18.8)
Gallbladder injury	3/108 (2.8)	1/16 (6.3)
Meningitis irritation	2/108 (1.9)	0
Secondary infections	10/108 (9.3)	2/16 (12.5)

^#^In this study 256 patients were confirmed as HAdV-7 positive infections among the 321 hospitalized patients by PCR and viral culture.

^*^In this study continuous variables were summarized as means (±SD) or medians (range).

**Table 2:** Laboratory and radiographic findings of the hospitalized patients from the two outbreaks†.

Variable	**Value**	
	**Xiangyang outbreak (n = 298)**†	**Jingmen outbreak (n** = **42)**
WBC count		
Median (range), 10^9^ cells/liter	10.4 (3.1–25)	10.5 (3.4–15.4)
>10 × 10^9^ cells/L, no. (%)	156 (52.3)	21 (50.0)
<4 × 109 cells/L, no. (%)	23 (7.7)	3 (7.1)
RBC count, median (range), 10^12^ cells/liter	4.46 (3.45–6.0)	4.31 (3.47–4.97)
Platelet count, median (range), 10^9^ cells/liter	213.2 (129–414)	223.5 (137–414)
Hemoglobin, median (range), g/liter	133 (98–312)	134 (102–156)
Glucose, >6.2 mmol/liter, no. (%)	76 (25.5)	9 (21.4)
Creatine Kinase, >200 U/liter, no. (%)	97 (32.6)	18 (42.9)
Creatine kinase MB fraction, >25 U/liter, no. (%)	85 (28.5)	18 (42.9)
Lactate dehydrogenase, >250 U/liter, no. (%)	39 (13.1)	5 (11.9)
Aspartate aminotransferase, >40 U/liter, no. (%)	43 (14.4)	6 (14.3)
Alanine aminotransferase, >40 U/liter, no. (%)	41 (13.8)	6 (14.3)
Total bilirubin, >17.1 μmol/liter, no. (%)	27 (9.1)	4 (9.5)
Uric acid, >416 umol/liter, no. (%)	17 (5.7)	2 (4.8)
Creatinine, >133 μmol/liter, no. (%)	13 (4.4)	2 (4.8)
Potassium		
median (range), mmol/liter	3.76 (3.0–5.61)	3.75 (3.18–4.57)
<3.5 μmol/liter, no./total no. (%)	32 (10.7)	8 (19.0)
Sodium, median (range), mmol/liter	138 (132–143)	138 (131–142)
Erythrocyte sedimentation rate, >15 mm/hr, no. (%)	79 (26.5)	20 (47.6)
C-reactive protein, >10 mg/liter — no. (%)	117 (39.3)	15 (35.7)
Abnormalities on electrocardiogram, no./total no. (%)	101/298 (33.9)	16/42 (38.1)
Sinus arrhythmia	60/101 (59.4)	11/16 (68.8)
High left ventricular voltage	27/101 (26.7)	5/16 (31.3)
Bundle branch block	3/101 (3.0)	1/16 (6.3)
T-wave change	7/101 (6.9)	0
Ventricular premature contraction	5/101 (5.0)	1/16 (6.3)
Abnormalities on chest radiograph and CT, no./total no. (%)	108/298 (36.2)	16/42 (38.1)
Local patchy shadowing	101/108 (93.5)	15/16 (93.8)
Ground-glass opacities	3/108 (2.8)	0
Pulmonary nodule	6/108 (5.6)	1/16 (6.3)
Mediastinal lymphadenectasis	5/108 (4.6)	2/16 (12.5)
Consolidation	4/108 (3.7)	1/16 (6.3)
Involvement of left lung	11/108 (10.2)	1/16 (6.3)
Involvement of right lung	41/108 (38.0)	4/16 (25.0)
Involvement of both lungs	56/108 (51.9)	11/16 (68.8)

^†^The clinical laboratory data on admission were available for 298 patients from the Xiangyang outbreak.

**Table 3:** Comparative genomic analysis of the outbreak strains with the reference strains and the local strain CDC228§.

**Region**	**Location**	**Gene**	**Nucleotide Change (amino acid substitution)**		
			**DG01_2011**	**CDC228**	**Outbreak strains**
ITR	518	inverted terminal repeat	Insertion of T	—	—
E1A	815	28 kDa protein	—	—	T→G (W→L)
	1444		—	—	A→G
IVa2	4133	—	—	—	C→T
E2B	5517	DNA polymerase	G→C (D→E)	—	—
	7494		—	—	G→T (L→I)
	9168	preterminal DNA-binding protein	—	—	C→T&
	9327		—	—	G→T&
L1	10698–10709	Virus-associated RNA II	—	—	Deletion of GTTGCGGTGTGC
	10734		—	—	G→A
	10816		—	Insertion of T	Insertion of T
	10830		G→A	—	—
L2	14594	Penton	T→C (V→A)	—	—
	14854		—	—	A→G (N→D)
L3	17871	Protein VI precursor	—	—	C→G (P→A)
L4	24298	hexon-assembly associated protein	C→T&	—	—
	24904		—	—	T→C&
E3	27799	16.1 kDa protein	—	—	C→T (P→S)
Non-coding Region	29950	—	—	—	Insertion of T
E4	32450	E4	—	—	T→C (N→D)
	33155	32 kDa protein	G→T (P→Q)	—	—
Non-coding Region	34904	—	—	—	G→A
ITR	35125	inverted terminal repeat	—	—	C→A

“–” means no change or not applicable.

^§^The reference genomes include the HAdV-7 strains DG01_2011 (KC440171) and 0901HZ/ShX/2009 (JF800905). Outbreak strains mean the nine HAdV-7 outbreak strains.

^&^Synonymous mutation.
