# Supplementary material for: Whole-genome Sequencing for Tracing the Transmission Link between Two ARD Outbreaks Caused by a Novel HAdV Serotype 7 Variant, China
Source: Sci Rep. 2015 Sep 4;5:13617. doi: 10.1038/srep13617 (PMC4559894; doi:10.1038/srep13617)
Supplement: Supplementary Information [file srep13617-s1.pdf]

Whole-genome Sequencing for Tracing the Transmission Link between Two ARD Outbreaks Caused by a Novel HAdV Serotype 7 Variant, China

Shaofu Qiu,[1*] Peng Li,[1*] Hongbo Liu,[1*] Yong Wang,[1*] Nan Liu,[1*] Chengyi Li,[1] Shenlong Li,[1] Ming Li,[2] Zhengjie Jiang,[3] Huandong Sun,[3] Ying Li,[2] Jing Xie,[1] Chaojie Yang,[1] Jian Wang,[1] Hao Li,[1] Shengjie Yi,[1] Zhihao Wu,[1] Leili Jia,[1] Ligui Wang,[1] Rongzhang Hao,[1] Yansong Sun,[1] Liuyu Huang,[1] Hui Ma,[4] Zhengquan Yuan,[1] and Hongbin Song[1]

Supplemental materials

Table S1. Primers used in this study for amplification and sequencing of possible pathogen target genes.

Table S2. The HAdV strains used in this study.

Table S3. Primers used in this study for gap closure of the genomes from the HAdV-7 outbreak strains.

Table S4. General characteristics of sequenced reads of the samples.

Table S5. Demographic and epidemiologic characteristics of the patients from the two HAdV-7 outbreaks.

Figure S1. Geographic distribution of the two HAdV-7 outbreaks in China. A and B represent the Xiangyang and Jingmen training base, respectively. The affiliated Xiangyang hospital was located in the Fancheng district, Xiangyang City, Hubei Province of China. This figure was generated using ESRI ArcGIS for Desktop v10.1 (ESRI Inc., http://www.esri.com).

Figure S2. The incidence rates of HAdV-7 infections among the outbreak patients, close contacts and local patients with ARD. The HAdV-7 strains were confirmed by real-time PCR, PCR sequencing and viral culture.

Table S1. Primers used in this study for amplification and sequencing of possible pathogen target genes.

Primer	Target gene	Sequence (5'-3')	Amplicon (bp)	Reference
adv-F	Hexon	ACATGCACATCGCCGG	145	1
adv-R		CGRTCSGTGGTCACATC		
adv-P		FAM-CGGGTCTGGTGCAGT-BHQ1		
adv-BL	Hexon	TTGACTTGCAGGACAGAAA	597	2
adv-BR		CTTGTATGTGGAAAGGCAC		
adv-FiBL	Fiber	TACCCCTATGAAGATGAAAGCA	1000	2
adv-FiBR		GGAGGCAAAATAACTACTCG		
adv-VA-F2	VA RNA	TACTGGCACTCCCGTCTCG	465	This study
adv-VA-R2		CCTCGCCCTCTTCCAACTC		
rRNA-F	16S rRNA	GTGCCAGCMGCCGCGGTAA	877	This study
rRNA-R		GACGGGCGGTGWGTRCA		

Table S2. The HAdV strains used in this study.

Isolate	Genotype	Serotype	Accession ID
HAdV isolate XY6 from Index case B of the Xiangyang outbreak	B	7	KJ019879
HAdV isolate XY1 from Index case A of the Xiangyang outbreak	B	7	KJ019880
HAdV isolate L34 from a quarantined patient of the Xiangyang outbreak	B	7	KJ019881
HAdV isolate K57 from from a quarantined patient of the Jingmen outbreak	B	7	KJ019882
HAdV isolate K22 from a quarantined patient of the Jingmen outbreak	B	7	KJ019883
HAdV isolate CDC228 from a sporadic patient in the local	B	7	KJ019884
HAdV isolate H12 from the Index case C from the Jingmen outbreak	B	7	KJ019885
HAdV isolate H18 from a hospitalized patient with severe pneumonia from the Jingmen outbreak	B	7	KJ019886
HAdV isolate JM11 from a quarantined patient of the Jingmen outbreak	B	7	KJ019887
HAdV isolate L14 from a hospitalized patient of the Xiangyang outbreak	B	7	KJ019888
HAdV type 3 strain GB	B	3	AY599834
HAdV B strain Guangzhou01	B	3	DQ099432
HAdV B strain Guangzhou02	B	3	DQ105654
HAdV B strain human/CHN/Ad4/2007/NEW [P3H3F7]	B	NEW [P3H3F7]	KF268311
HAdV type 7	B	7	AC_000018
HAdV type 7 vaccine strain	B	7	AY495969
HAdV type 7 strain Gomen	B	7	AY594255
HAdV type 7 vaccine strain	B	7	AY594256
HAdV type 7 strain NHRC 1315	B	7	AY601634
HAdV 7 VA RNA region, strain hAdVi/Caen.FRA/11.07(5043)	B	7	FR718945
HAdV 7 strain GZ08	B	7	GQ478341
HAdV 7 strain gz07	B	7	HQ659699
HAdV 7 strain 0901HZ/ShX/CHN/2009	B	7	JF800905
HAdV 7d2 strain FS2154	B	7	JN860677

HAdV B strain human/USA/ak35_AdV7d2/2006/7[P7H7F7]	B	7	JX423383
HAdV B strain human/ARG/ak38_AdV7h/2003/7[P7H7F7],	B	7	JX423386
HAdV B strain human/USA/ak39_AdV7d2/1997/7[P7H7F7]	B	7	JX423387
HAdV B strain human/USA/ak40_AdV7b/1997/7[P7H7F7]	B	7	JX423388
HAdV 7 isolate CQ1198	B	7	JX625134
Human adenovirus 7 strain human/CHN/DG01/2011/7[P7H7F7]	B	7	KC440171
HAdV B strain human/USA/UFL_Adv7d2-2/unknown/7[P7H7F7],	B	7	KF268117
HAdV B strain human/USA/CL_44/1988/7[P7H7F7]	B	7	KF268125
HAdV B strain human/USA/UFL_Adv7h/2005/66[P7H7F3]	B	7	KF268126
HAdV B strain human/USA/CL_43/1988/7[P7H7F7]	B	7	KF268134
HAdV B strain human/USA/UFL_Adv7d2-3/unknown/7[P7H7F7]	B	7	KF268135
HAdV B strain human/CHN/DG/2011/7[P7H7F7]	B	7	KF268314
HAdV B strain human/CHN/a238/2011/7[P7H7F7]	B	7	KF268316
HAdV type 11 strain Ad11p Slobitski	B	11	AY163756
HAdV 14 strain HAdV14-BJ430/CHN2010	B	14	JN032132
HAdV 14 isolate CHN 2012	B	14	JX892927
HAdV type 16 strain ch. 79	B	16	AY601636
HAdV type 21 strain AV-1645	B	21	AY601633
HAdV type 34 strain Compton	B	34	AY737797
HAdV type 35 strain Holden	B	35	AY128640
HAdV type 50 strain Wan	B	50	AY737798
HAdV 55 strain QS-DLL	B	55	FJ643676
HAdV B isolate Human/CHN/BJ01/2011/55/P14H11F14	B	55	JX491639

Table S3. Primers used in this study for gap closure of the genomes from the HAdV-7 outbreak strains.

Primer	Sequence (5'-3')	replicon (bp)
CDC228_1F	GGAGAACCTGCACTACACCT	145
CDC228_1R	TTTAAGGGTGGTGTGCAGGA	
CDC228_2F	CAGGGTTACTCTGCGAAACG	209
CDC228_2R	GGTAGGAGAGGTAGTTGGCC	
XY1_1F	ACCACACTACCTCCTATGCA	263
XY1_1R	ATACAGTCTGGGCTTGCAGT	
XY1_2F	CCTTCAACCCTGTCTACCCC	245
XY1_2R	AGTCTTAACGAGTGGTGTGG	
L34_1F	AATTAATCGCCAGGGTTGCC	219
L34_1R	TTTGTGGCTGTCGTTGTAGC	
H12_1F	CAGGGTTACTCTGCGAAACG	209
H12_1R	GGTAGGAGAGGTAGTTGGCC	
JM11_1F	ACCACACTACCTCCTATGCA	263
JM11_1R	ATACAGTCTGGGCTTGCAGT	

Table S4. General characteristics of sequenced reads of the samples.

Sample	No. of raw reads	No. of viral reads	Expected coverage
CDC228	121,882	109,743	158
H12	159,250	138,165	137
H18	123,492	64,031	57
JM11	123,298	82,930	114
K22	122,774	76,930	61
K57	136,722	106,110	62
L14	142,878	39,820	23
L34	123,808	75,176	63
XY1	127,836	109,492	124
XY6	106,966	103,586	65

Table S5. Demographic and epidemiologic characteristics of the patients from the two HAdV-7 outbreaks.

Characteristic	Value	
	Xiangyang outbreak (n=858)	Jingmen outbreak (n=88)
Age, mean, yr	19.7±1.9	20.1±1.9
Male sex — no. (%)	773 (90.1)	88 (100)
Incidence rate, no./total no. (%)	858/1290 (66.5)	88/865 (10.2)
Smoking status — no. (%)		
Current smoker	65 (7.6)	8 (9.1)
No history of smoking	747 (87.1)	77 (87.5)
Unknown/missing	46 (5.4)	3 (3.4)
Underlying condition — no. (%)		
Any	82 (9.6)	15 (17.0)
Allergy to antibiotics	52 (6.1)	7 (8.0)
Excimer laser eye surgery	10 (1.2)	2 (2.3)
Tonsillectomy	4 (0.5)	2 (2.3)
Pneumonia	5 (0.6)	0
Chronic gastritis	3 (0.3)	1 (1.1)
Chronic rhinitis	2 (0.2)	1 (1.1)
Appendicitis	2 (0.2)	0
Hernia surgery	0	1 (1.1)
Hepatitis B infection	0	1 (1.1)
Hyperthyroidism	1 (0.1)	0
Hydrocele surgery	1 (0.1)	0
Otitis media	1 (0.1)	0
Hemorrhoid surgery	1 (0.1)	0
No underlying conditions	730 (85.1)	70 (79.5)
Unknown/missing	46 (5.4)	3 (3.4)

References

[1] Ebner, K., Suda, M., Watzinger, F., & Lion, T., Molecular detection and quantitative analysis of the entire spectrum of human adenoviruses by a two-reaction real-time PCR assay. *J Clin Microbiol* 43 (7), 3049-3053 (2005).

[2] Madisch, I., Harste, G., Pommer, H., & Heim, A., Phylogenetic analysis of the main neutralization and hemagglutination determinants of all human adenovirus prototypes as a basis for molecular classification and taxonomy. *J Virol* 79 (24), 15265-15276 (2005).

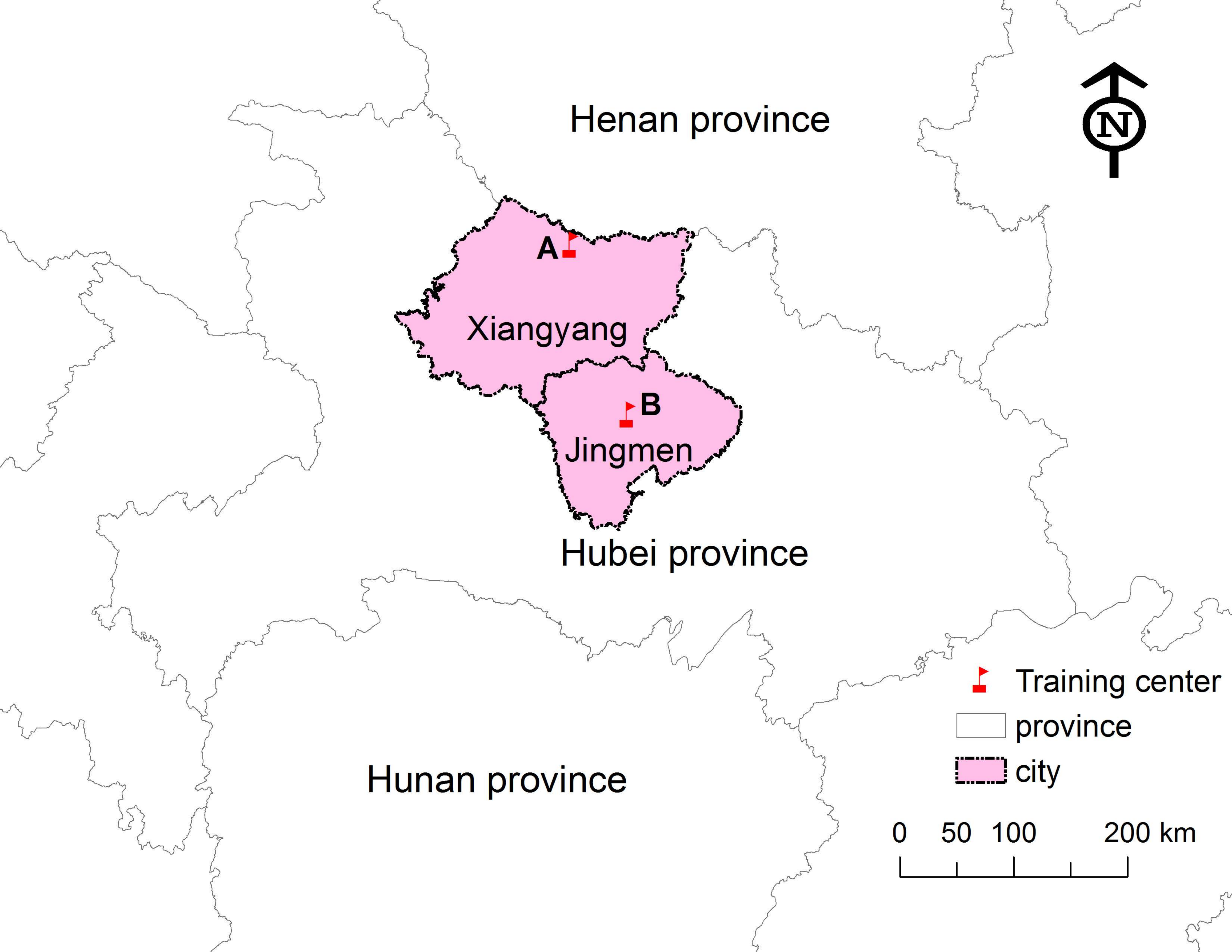

Henan province
N
A
Xiangyang
B
Jingmen
Hubei province
Hunan province
Training center
province
city
0
50
100
200 km

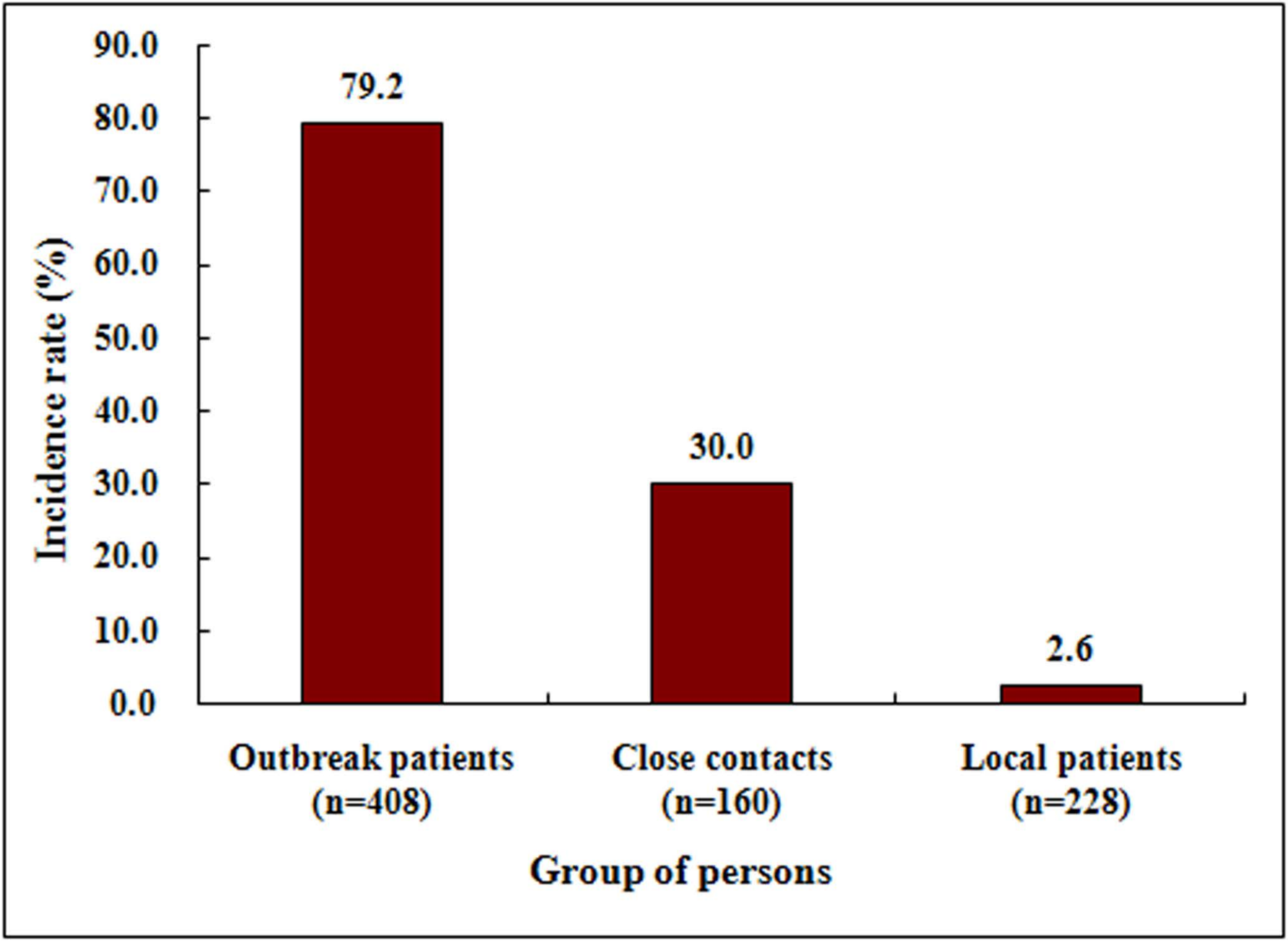

Incidence rate (%)
90.0
80.0
70.0
60.0
50.0
40.0
30.0
20.0
10.0
0.0
79.2
30.0
2.6
Outbreak patients (n=408)
Close contacts (n=160)
Local patients (n=228)
Group of persons
